# Towards holistic colony feeding: Effects of vitamin supplementation on summer and winter honey bee workers, *Apis mellifera* L

**DOI:** 10.1371/journal.pone.0328626

**Published:** 2025-08-28

**Authors:** Andrew F. Brown, Leah Guillaume-Gentil, Johanna Hehl, Stefan Niederer, Gina Retschnig, Peter Neumann

**Affiliations:** Institute of Bee Health, Vetsuisse Faculty, University of Bern, Bern, Switzerland; Universidade de São paulo, BRAZIL

## Abstract

In managed western honey bee, *Apis mellifera*, colonies, vitamins are often neglected when it comes to SI feeding despite their importance for health. Moreover, the potential links of vitamin feeding to longevity, food consumption and adult dry weight are poorly understood. Finally, comparative nutritional studies of short-lived summer workers and long-lived winter workers are currently lacking. Here, in a fully-crossed design, multivitamin supplementation of vitamins A, D, E, K, C, B1, B2, B3, B5, B6, B8, B9 and B12 (N = 13), titrated in three different dosages were fed to summer and winter workers in two trials. In standard laboratory hoarding cages, experimental workers were assigned one of eight treatments: Sucrose, Sucrose + Pollen, Vitamin 1, Vitamin 1 + Pollen, Vitamin 2, Vitamin 2 + Pollen, Vitamin 3, Vitamin 3 + Pollen (N = 8 treatments, 26 workers/cage, N = 64 cages/phase, N = 3328 total workers). Regardless of season, significant consumption polynomial trends were revealed; however, no significant differences in sucrose and pollen consumption were found. Likewise, none of the used multivitamin dosages did significantly improve any of the measured parameters. On the other hand, *ad libitum* access to pollen consistently increased both weight and lifespan, reinforcing pollen as critical to honey bee health. Additionally, the data clearly show that summer and winter workers bees display very similar significant patterns of dry weight and longevity. In particular, summer bees lived longer than winter ones. In light of well-known differences between summer and winter workers in the field, in particular longevity, these results are unexpected. Therefore, improved laboratory settings for honey bee s seem to be required to obtain more biologically relevant data and ultimately improve managed *A. mellifera* health.

## Introduction

Global losses of managed western honey bee, *Apis mellifera* colonies, particularly in brood-free periods (i.e., dry-season, winter) continue to persist [1–3]. Hence, finding robust and easy-to-employ solutions for successful hive management is key. Extensive research has revealed commonalities linked to colony failure, such as climate change [4], landscape change [5], invasive species [6,7], opportunistic pathogens [8], and nutrient deficiencies [9,10]. Indeed, supplementation of macronutrients (e.g., proteins, sugar) to enhance colony strength is standard practice [11], however, there is still a need for greater focus on proper micronutrient dosing [12], and furthermore, nutrition-based comparative studies from summer bees replicated in winter bees are currently lacking. Given that summer bees do the primary food preparation for their winter bee counterparts, finding supplements that are well-tolerated by summer bees with potential downstream benefits to winter bees seems to be a well-reasoned starting point.

Geographic location of *A. mellifera* colonies dictate seasonal cycles of brood and brood-free periods (e.g., spring/summer seasons [13] vs. winter/dry seasons [14]), resulting in short-lived summer and long-lived winter workers with distinct physiological and ecological traits [15]. Summer bees live ≈4–6 weeks [15], with shortened lifespan linked to factors such as energy expenditure activities (e.g., foraging, [16]), juvenile hormone and/or vitellogenin titers [17], and possibly demands of maintaining colonies (i.e., brood-care and foraging, [18]). Winter bees, on the other hand, can live > 200 days [19,20]. These long-lived workers, with reduced foraging and brood care needs [15], must sustain themselves on nutrients found in the colony, combined with accumulated nutrients stored in forms such as glycogen and triglycerides (TG) [18,21]. TG’s are of exceptional interest in the context of winter bees given their dense caloric properties [22], and it is well known that winter bees physiologically have significant higher TG content than their summer bee counterparts [23,24]. Indeed, low levels of TGs in early-stage dipterans translates to curtailed late-stage body size and reduced fecundity [25], and lower lifespan in honey bees [26]. Furthermore, hemolymph-vitellogenin levels, made from stored TGs, are continuously elevated over an extended duration in winter bees, and act as a reliable marker for extended lifespan [17]. Given that TGs are directly related to the nutritional status of insects [21], together with the knowledge that macro- and micronutrients work in parallel (e.g., B-vitamins, such as biotin, are necessary for TG metabolism [27,28], determining the tolerance of summer bees to micronutrient-fortified supplements, and evaluating the potential long-term advantages for winter bees, would provide crucial insights to apiculturists.

Pollen has repetitively been shown as being advantageous to honey bee s [10,29], from early apiculture research underscoring the essential roles for nurse bees and emerging adults [30], to more contemporary evidence of pollen on health indicators such as bodyweight and longevity [11,31]. Indeed, pollen is the main source of protein for bees [24,29], and in winter bees, ample protein in formative stages (i.e., first 12 days post-emergence) is necessary to increase hemolymph-protein ratios high enough to make the transition from summer to winter worker [18]. Furthermore, cost-benefit analyses of nonpollen-based protein sources (e.g., soy, yeast) have been carried out and demonstrated to increase hemolymph-protein levels [32], yet true-pollen appears to consistently perform better [33]. Although a “fully-balanced” artificial diet in times of need for *A. mellifera* has yet to be established, decades of detailed studies have focused on the macronutritional needs of honey bees [34], and as such, evidence-based macronutrient supplementation guidelines and products are readily available for beekeepers [35,36]. In addition to macronutrients, pollen is also rich in micronutrients (e.g., fat- and water-soluble vitamins [37], which are only recently gaining attention in honey bee supplementation research.

The role of micronutrient (i.e., vitamin) supplements in *A. mellifera* health has also been investigated. Early research established that pyridoxine (B6) is essential for brood rearing [38,39], and shortly after, adult workers fed supplements containing vitamins A, D, E, and K doubled colony-brood production [40]. Vitamin C, although not labeled as essential for bees, has been shown to significantly reduced oxidative stress and enhance brood rearing in free-flying colonies [41] while in once instance significantly mediating winter colony losses [42]. Recently, B-vitamins have shown improvement in colony strength assessments (i.e., open/sealed brood, food reserves, adult populations numbers) and decrease viral and pathogenic loads in field colonies [43]. Further, B-vitamins operate simultaneously with macronutrients, acting as obligate coenzymes for fat, protein, and carbohydrate metabolism [44]. Nonetheless, upper-limits of B-vitamins appear to exist [12], and established micronutrient-guidelines are persistently absent.

Longevity and bodyweight have been repetitively used as validated *A. mellifera* worker health indicators [18,45–47]. Indeed, the relationship between body weight and longevity allows researchers to predict the outcomes of different interventions and management practices on colony health [48,49], which later aid in the development of effective beekeeping strategies. Knowing that nutrition can affect all stages of bee development and survival outcomes [50,51], formulating agreed-upon nutrition guidelines that support longevity and bodyweight is of great importance [52].

Comparative studies of winter and summer bees are critical for understanding seasonality and *A. mellifera* colony dynamics. Generally speaking, foraging summer bees are the primary source for ensuring a colony’s access to nutrients, and furthermore, they prepare the predominant portion of food for winter bees [15,53]. Given the common-practice of supplementing summer bees, knowing if consequences from the supplements are carried over to winter bees would be a beneficial stepping stone for managed colonies. Indeed, a plethora of *A. mellifera* research has focused on summer bees, in particular due to their higher degree of interaction with environmental factors, making them ideal subjects for studying variables such as xenobiotic-exposure impacts [54] and pathogen-induced stress studies [55]. Additionally, the higher egg laying of queens in summer [15,20] facilitates immediate access to newly emerged workers, data collection, and easily observable results in experimental settings. However, colony loss in brood-free seasons remains particularly problematic [3,56], making an argument for more controlled laboratory studies of winter bees to enhance colony resilience [14] and promote better management strategies.

Here, we use a fully-crossed hoarding cage experiment to investigate sucrose and pollen consumption habits, dry body weight, and longevity in summer and winter bees and three different multivitamin dosages ( [12,40,42] Table 1). Given the aforementioned tandem roles macro- and micronutrients play in *A. mellifera* health, we would anticipate synergistic improvements from the vitamins and pollen in the measured parameters. Lastly, given the extensive evidence that winter and summer bees have different lifespans, we hypothesize that winter bees would indeed live longer.

**Table 1:** Vitamin dosages (N = 3) used to make the multi-vitamin solutions (N = 13 total vitamins). Vitamins A, D, E, K, C, B1, B2, B3, B5, B6, B8 & B9 and their respective dosages per vitamin, expressed in mg/L, is provided.

Vitamin	Dose 1 (mg/L)	Dose 2 (mg/L)	Does 3 (mg/L)	Reference
A	0.004	0.04	0.4	Herbert et al., 1978
D	0.004	0.04	0.4	Herbert et al., 1978
E	0.004	0.04	0.4	Herbert et al., 1978
K	0.004	0.04	0.4	Herbert et al., 1978
C	1.8	3.6	7.2	Farjan et al., 2012
B1 (Thiamine)	2	4	8	Brown et al., 2021a
B2 (Riboflavine)	5	10	20	Brown et al., 2021a
B3 (Nicotinamide)	50	100	200	Brown et al., 2021a
B5 (Pantothenic Acid)	12.5	25	50	Brown et al., 2021a
B6 (Pyridoxine)	5	10	20	Brown et al., 2021a
B8 (Biotine)	0.1875	0.375	0.75	Brown et al., 2021a
B9 (Folic Acid)	0.5	1	2	Brown et al., 2021a
B12 (Cyanocobalamin)	0.00625	0.0125	0.025	Brown et al., 2021a

## Materials and methods

Two hoarding cage experiments were performed at the Institute of Bee Health (Bern, Switzerland, 46°55’48.6“N 7°25’23.2”E). The first phase was conducted from May – August 2021 (local spring/summer), and the second from September – December 2021 (local autumn/winter). In both instances, experimental workers were obtained from four local, unrelated and queenright *Apis mellifera mellifera x carnica* colonies (N = 4 Dadant hives). From each colony, two brood frames pre-verified to have final-stage pupae were selected, brushed clean, and incubated until adult emergence (34.5°C, > 60% Relative Humidity RH, [57]. After 48 hours, all newly emerged workers were mixed in a single container, homogenizing colony genetics, and randomly placed in 100 cm^3^ clear polystyrol cages (N = 2 phases, N = 64 cages/phase, 26 workers/cage, N = total workers 1664/phase, N = 3328 total workers, [57]. Post-cage assignment, each cage was designated one of eight treatments (N = 8 treatments, Table 2), with eight replicates per treatment (N = 8), and maintained in a Memmert^©^ HPP 750 climate chamber, set to 30°C and >60% RH [57]. Mortality checks were done once every 24 hours until the last remaining bee died.

**Table 2:** Treatment groups (N = 8) assigned to experimental A. mellifera adult workers: Sucrose, Sucrose + Pollen, Vitamin 1, Vitamin 1 + Pollen, Vitamin 2, Vitamin 2 + Pollen, Vitamin 3, Vitamin 3 + Pollen. Treatment names, total number of workers/trial (Summer & Winter) and global totals are displayed.

Group	Treatment	Replicates	N total summer workers	N total winter workers	
1	Sucrose	8	208	208	
2	Sucrose + Pollen	8	208	208	
3	Vitamin 1	8	208	208	
4	Vitamin 1 + Pollen	8	208	208	
5	Vitamin 2	8	208	208	
6	Vitamin 2 + Pollen	8	208	208	
7	Vitamin 3	8	208	208	
8	Vitamin 3 + Pollen	8	208	208	
**TOTAL**			**1664**	**1664**	**3328**

### Dietary treatments

All liquid diets were provided to experimental workers via 5 ml syringes vertically placed through pre-made inserts on each cage [57]. Fresh sucrose solution (50% w/v) was made on a weekly basis with sterilized tap water, and vitamin isolates were individually solved in the sucrose solution to desired concentrations (Table 1), or the solution was left blank (sucrose only, negative control). All solutions were stored at 4^o^C until use. Groups 2, 4, 6, and 8 (Table 2) were given additional *ad libitum* access to locally sourced poly-floral pollen (Swiss Pollen, Bienen Roth), provided horizontally in modified 1.5mL Eppendorf tubes with a clipped tip and inserted through the side of each cage [57]. Prior to introduction, pollen was mixed to a 10:1 ratio with distilled water [57], made fresh on a weekly basis, and stored at -20^o^C until use. Lastly, all syringes and pollen tubes were changed with fresh ones on a bi-weekly basis, limiting mold and/or bacterial fermentation.

### Vitamin solutions

Vitamin concentrations were prepared by dissolving vitamin isolates into solvents to create high concentrated stock solutions [12]. The vitamins were purchased from Hänseler AG (B1, B2, B3, B5, B6, B8), PureBulk (B9) and Sanofi Chimie BP (B12), and Thermo Fisher Scientific (A, C, D, E, and K). Once prepared, the vitamins were individually pipetted into sucrose solution (50% (w/v)) to desired multivitamin treatment concentrations (Table 1) and stored at 4°C until use. All vitamin stock solutions were freshly prepared on a weekly basis.

### Food consumption

Pollen and sucrose consumption were recorded per cage/treatment once every 24 hours for 24 days. Briefly, both syringes and pollen tubes were weighed separately on a Mettler Toledo PR5003 scale (precision 10^-3^g), and the weight difference from the preceding day was calculated. Lastly, the final weight difference was divided by the number of workers present in each cage at that time point, yielding a food consumption average/day/cage. All consumption values were adjusted for evaporation and/or mechanical loss [58].

### Dry body weight

Dry weight was chosen to exclude water weight from each sample. Briefly, weight was taken 1- and 2- weeks post-trial commencement (N = 2 Sample increments). For each sampling, three randomly selected workers were removed from each cage, put in individually labeled Eppendorf^©^ tubes, and fresh weight was taken on a Mettler AT 400 scale (precise to 10^-4^g). All tubes were subsequently transferred to an incubator (Memmert UM 100), set at 45°C, and removed and re-weighed every 24 hours until all successive measures were constant (±0.001g, [12,59].

### Statistical analyses

Statistical analyses were performed with R (version 4.3.2, [60]). Data distributions were visually checked (e.g., residual error, [61]) combined with Jarque-Bera normality tests when appropriate [62].

### Food consumption

#### Sucrose consumption.

For sucrose consumption, a least-squares regression model (lm) with square root transformed data was used with “consumption” dependent on “treatment” and “time” (modeled as a 2^nd^ degree polynomial). Parametric pairwise testing, based on the linear model output, was done with the “multcomp” library [63] using the *glht* command with “Holm” corrected Ps [64].

#### Pollen consumption.

Pollen consumption data, with untransformed data, were analyzed using the same aforementioned steps for sucrose consumption.

#### Protein-to-carbohydrate.

Ratios of protein-to-carbohydrates (P:C) through time were calculated and also modeled as 2^nd^ degree polynomials in least-squared regression model (lm), with an interaction term between “time” and “season” to determine whether seasoned influenced protein-to-carbohydrate consumptions.

#### Dry body weight.

For the body weight analysis, untransformed data were used two separate least-squared regression models (lm). The first model was done with “weight” dependent on “time”, and the second with “weight” dependent on “treatment”. Non-parametric comparisons between all treatments were done with a pairwise Wilcoxon test, with “Holm” corrected Ps [64].

#### Survival.

The survival analysis was done with the “survival” [65,66] and “surminer” [67]. The *Surfdiff* function was used to calculate survival curves and perform log-rank (rho = 0) and chi-squared tests. The *pairwise_survdiff* function was used to for pairwise survival testing, and a Bonferroni p-value adjustment was applied [68].

### Declarations

Ethics approval: No approval of research ethics committees was required to accomplish the goals of this study because experimental work was conducted with an unregulated invertebrate species.

## Results

### Food consumption

Food consumption was measured for 24 days, and sucrose consumption ranged from 4.05–80.61 mg/day, with an overall mean of 31.28 and standard deviation of ±11.23 mg (summer bees, S1 Table) and 4.21–93.74 mg/day, with an overall mean of 32.26 and standard deviation of ±12.68 mg (winter bees, S1 Table). Pollen consumption ranged from 0.05–7.8 mg/day, with an overall mean of 2.47 mg and standard deviation of ±1.84 mg (summer bees, S1 Table) and 0.11–11.11 mg/day, with an overall mean of ±2.55 mg and standard deviation of 1.95 mg (winter bees). In both instances, the data (i.e., residual error) fit normality assessments (Jarque-Bera Normality Test, Ps > 0.05).

**Fig 1:**
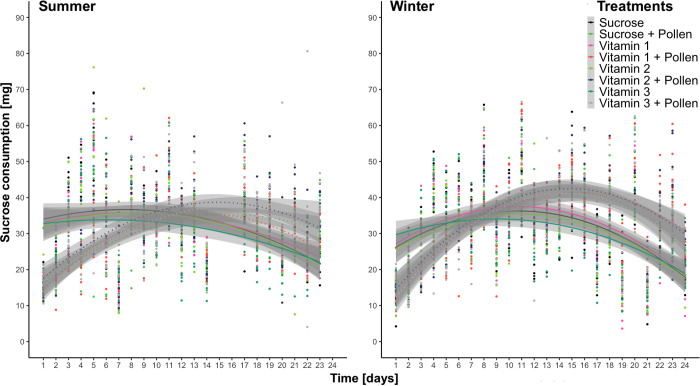
Scatter plot of sucrose consumption [mg] through time fit with least-squared regression lines (2^nd^ degree polynomial) from adult summer (left) and winter (right) Apis mellifera workers from eight treatments: Sucrose, Sucrose + Pollen, Vitamin 1, Vitamin 1 + Pollen, Vitamin 2, Vitamin 2 + Pollen, Vitamin 3, Vitamin 3 + Pollen. Each line and color represent one of eight treatments, shaded with their respective 95% confidence interval. Dashed lines represent groups supplemented ad libitum pollen. No significant differences in sucrose consumption habits were found (Tukey comparison of means, Holm adjusted Ps, Ps > 0.05).

Significant fluctuations through time (Fig 1) were found in sucrose consumption, where in each trial, an initial significant increase in consumption was observed, followed by a significant decrease (lm, S2 Table, Ps < 0.05). Additionally, *ad libitum* access to pollen resulted in general significant decreases in sucrose consumption for summer bees when compared to the negative control “Sucrose” (lm, S2 Table, Ps < 0.05), however, it was the predicted to be overall statistically higher for winter bees when compared to the negative control “Sucrose” (lm, S2 Table, Ps < 0.05). In the summer trial, no significant differences were found between treatment groups (lm, Tukey multiple mean comparison, Holm adjusted Ps, all Ps > 0.05). In the winter trial, with the exception of “Vitamin 3” consuming the statistically least amount (lm, Tukey multiple mean comparison, Holm adjusted Ps, all Ps > 0.05), no statistical differences were found between the other treatment groups (lm, Tukey multiple mean comparison, Holm adjusted Ps, all Ps > 0.05).

**Fig 2:**
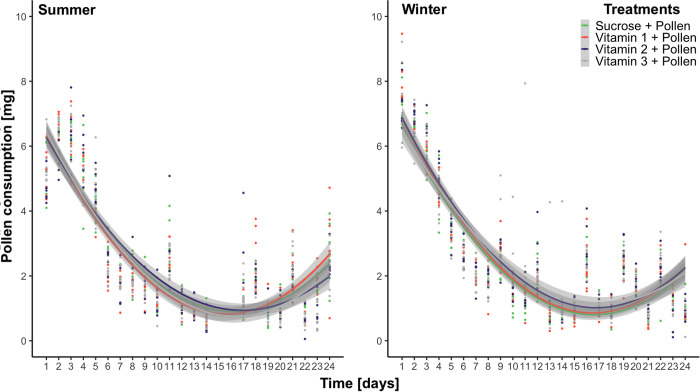
Scatter plot of pollen consumption [mg] through time fit with least-squared regression lines (2^nd^ degree polynomial) from adult summer (left) and winter (right) Apis mellifera workers from eight treatments: Sucrose, Sucrose + Pollen, Vitamin 1, Vitamin 1 + Pollen, Vitamin 2, Vitamin 2 + Pollen, Vitamin 3, Vitamin 3 + Pollen. Each line and color represent one of eight treatments, shaded with their respective 95% confidence interval. No significant differences in pollen consumption habits were found (Tukey comparison of means, Holm adjusted Ps, Ps > 0.05).

**Fig 3:**
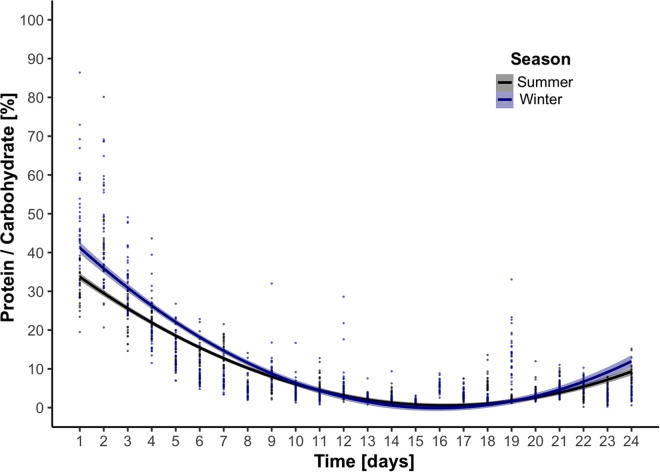
Scatter plot of pollen-to-carbohydrate ratio [%} through time fit with least-squared regression lines (2^nd^ degree polynomial) from adult summer (black) and winter (blue) Apis mellifera workers. Each line and color represent one season, shaded with their respective 95% confidence interval.

In the case of pollen consumption, similar significant fluctuations in consumption habits through time were also found (lm, Ps < 0.05, Fig 2), however, the linear relationships were inversely symmetrical to that of sucrose consumption, translating to an initial decrease in pollen consumption, followed by a significant increase. No significant differences were found between treatments (lm, Tukey multiple mean comparison, Holm adjusted Ps, all Ps > 0.05). Protein to carbohydrate (P:C) ratios were also calculated, and likewise, also showed significant fluctuations through time (Fig 3). Lastly, a significant interaction between time and season was found (S2 Table), highlighting “winter” bees as having a higher P:C ratio than “summer” bees (lm, S2 Table), which is visually intriguing in the first 8-days (Fig 3).

### Dry body weight

Dry weight ranged from 17.30–60.40 mg, with an overall median of 32.05 mg and IQR of 28.96–35.05 mg (summer bees, S3 Table) and 18.30–64.20, with an overall median of 31.05 and IQR of 27.53–34.12 mg (winter bees, S3 Table). In both trials, the data (i.e., residual error) did not pass normality assessments (Jarque-Bera Normality Test, Ps < 0.05).

**Fig 4:**
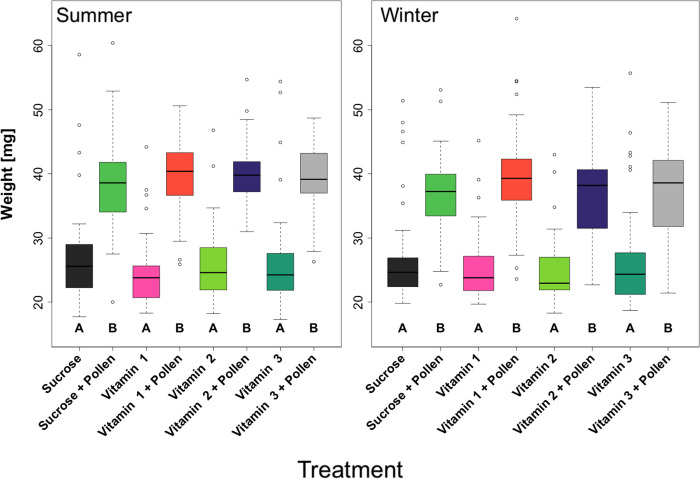
Boxplots from adult summer (left) and winter (right) Apis mellifera workers from eight treatments: Sucrose, Sucrose + Pollen, Vitamin 1, Vitamin 1 + Pollen, Vitamin 2, Vitamin 2 + Pollen, Vitamin 3, Vitamin 3 + Pollen. (N = 8 treatments, N = 48 workers/treatment group/season). Each color and boxplot represent a different treatment. Minimum and maximum values, as well as lower quartiles, medians, and upper quartiles are displayed. Significant differences between treatments are indicated by letters based on pairwise Wilcoxon tests, with Holm adjusted Ps, (Ps < 0.05).

In each case, the weights between the experimental workers did not significantly differ between the two sample periods (lm, Ps > 0.05), justifying the pooling of data. Furthermore, regardless of season, experimental workers given *ad libitum* access to pollen consistently had significantly higher body weights (Fig 4, pairwise Wilcoxon test, Holm adjusted Ps < 0.05, Letter B) than their non-pollen supplemented counterparts (Fig 4, pairwise Wilcoxon test, Holm adjusted Ps < 0.05, Letter A).

### Survival

**Fig 5:**
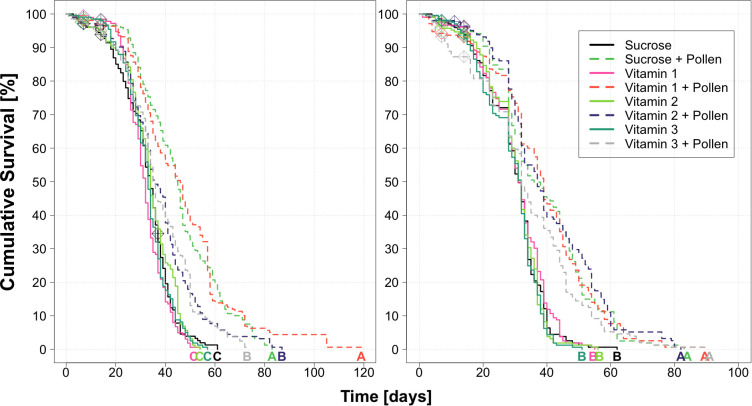
Kaplan-Meier survival curves from adult summer (left) and winter (right) Apis mellifera workers from eight treatments: Sucrose, Sucrose + Pollen, Vitamin 1, Vitamin 1 + Pollen, Vitamin 2, Vitamin 2 + Pollen, Vitamin 3, Vitamin 3 + Pollen. (N = 208 workers/treatment group/season, N = 8 replicates/treatment, N = 26 bees/treatment, N = 1664 total workers/season. Each line and color represent a different treatment, and dashed lines indicate groups supplemented ad libitum pollen. Significant differences between survival outcomes of the experimental workers are indicated by letters based on log rank tests and Bonferroni Ps (Ps < 0.05).

Life expectancy from the eight treatment groups ranged between 1–119 days, with an overall median of 32 days and IQR of 15–42 days (summer bees, S4 Table), and 1–90 days, with an overall median of 29 days and IQR of 14–39 days (winter bees, S4 Table). For the summer trial, highest survival was observed in the groups Sucrose + Pollen and Vitamin 1 + Pollen (Fig 5 “summer”, Kaplan Meier, Log Rank test, Ps < 0.05, Letter A) followed by Vitamin 2 + Pollen and Vitamin 3 + Pollen (Fig 5 “summer”, Kaplan Meier, Log Rank test, Ps < 0.05, Letter B). All other groups, in the absence of pollen, had significantly lower lifespans: vitamin groups 1, 2, and 3, as well as the negative control Sucrose (Fig 5 “summer”, Kaplan Meier, Log Rank test, Ps < 0.05, Letter C). For the winter trial, all groups supplemented with *ad libitum* pollen lived statistically the longest (Fig 5 “winter”, Kaplan Meier, Log Rank test, Ps < 0.05, Letter A), and all other groups, in the absence of pollen, had statistically similar outcomes as the negative control “Sucrose” (Fig 5 “winter”, Kaplan Meier, Log Rank test, Ps < 0.05, Letter B).

Lastly, given no positive effect from treatments were found, comparisons between the controls from both seasons was done, and showed that both “Summer” groups had higher longest predicted lifespans than their winter bee equivalents (S1 Fig, Kaplan Meier, Log Rank test, Ps < 0.05, Letters A-D, S4 Table).

## Discussion

The data here surprisingly demonstrate that, despite the known ecological differences between short-lived summer bees and long-lived winter bees, similar predictions for bodyweight and food consumption amounts (mg) were found. Unexpectedly, differences in longevity were uncovered between the two seasons, where “summer” bees actually lived longest, highlighting a call for changing laboratory protocols to better mimic field conditions. In every instance, pollen was the most significant beneficial factor for all measured parameters when compared to the negative control “sucrose”. Furthermore, the P:C data add proxy-evidence that a separation of “winter” and “summer” was successfully tested achieved. On the other hand, none of the multivitamin dosages proposed here independently improved dry body weight and longevity, underscoring that the current dosages can still be optimized for these parameters. Considering the ever-increasing challenges managed honey bee colonies are facing, we suggest that fine-tuning standardized protocols of *A. mellifera* laboratory research is imperative in future studies in order to translate true impacts from lab-results to field colonies.

The sucrose and pollen consumption data interestingly show that each and every treatment followed the same statistically significant trends of either increasing initial consumption (e.g., carbohydrates), yet followed by a decrease after ≈14 days, or in the case of pollen, the symmetrical inverse. The uniformity in consumption patterns indicates that while vitamins are indeed essential for insects [10,29,69], their presence in the diets did not significantly alter consumption behavior in either season, thus ruling out these variables as influencing the bodyweight and longevity results. On the other hand, the P:C data presented here add proxy-evidence that we indeed have a separation of “summer” and “winter” bees in our two trials. As alluded to in the introduction, protein status acts as a clear variable in prediction *A. mellifera* lifespan [70], and ample protein buildup in workers ≤12 days old is necessary to reach hemolymph-protein ratios high enough to make the transition from “summer” to “winter” bee [18]. Furthermore, this period of development, with particular interest in vitellogenin levels (i.e., marker for longevity and winter bees, [17], is indeed shown to take approximately 2 weeks [71–73], as our Fig 3 would suggest. Furthermore, the data suggest that P:C ratios must intrinsically be regulated by bees [74]. Previous studies have shown that excess hemolymph-protein levels lead to elevated mortality [75,76], however unlike published experiments where protein was artificially elevated in single-choice assays to explore upper-limits of P:C ratios [76], here the workers were presented with free-choice assays between sucrose solution and pollen tubes. Fig 3 clearly shows significant up-and-downward (i.e., polynomial) trends, with the workers likely avoiding over-consumption of proteins. Future studies coupling physiological measurements, such as hypopharyngeal gland measurements [74], fat-body content [24], vitellogenin and/or juvenile hormone titers [17], would be beneficial to correlating our P:C data to other verified “winter” and “summer” bee measurements.

The body weight and longevity results of this study underline a significant positive effect from *ad libitum* access to pollen. Abundant published data confirm the positive benefits of pollen [77,78], such as increased longevity [12,45,79] and adult dry weight [11,31,80]. Such results are expected, and likely tied to the innate macro- and micronutrients found in pollen [10,37]. Furthermore, protein-replacements are very promising [32], and recent findings from amino-acid mixes based on amino-acid profiles from pollen show positive results for boosting honey bee immunity [81], but nonetheless, wild poly-floral sourced pollen still appears to be the optimal choice when available [33,82]. Here, the experimental workers were provided with a locally-sourced poly-floral pollen (Swiss Pollen, Bienen Roth), cohering part of our results with above-mentioned studies. However, our hypothesis that the multivitamins mixed in the sucrose solution would play a synergistic role when coupled with pollen cannot be accepted, as the positive control “Sucrose + Pollen” performed equally as well in both weight and lifespan predictions. On the other hand, the longevity date exemplifies an inherent issue stemming from the standardized hoarding cage conditions used here [57], which clearly altered the expected lifespan of ≈200 days [19,20,83] for winter bees versus ≤90 days we achieved. As discussed in previous work of ours from caged winter bees [12], this underscores the challenge of applying lab trial observations to natural hive scenarios, and already recognized phenomenon in the bee research [84].

Research into *A. mellifera* nutritional needs has highlighted the significant impact vitamins can have on their health and productivity. For instance, Farjan and colleagues (2012) demonstrated that vitamin C induced oxidative-stress resistance in larvae, critical to combating exogenous stress factors (e.g., *Varroa destructor*). Moreover, vitamin-rich laden supplements can improve overall colony health and longevity [43], underscoring the importance of such micronutrients, and the on-going need of a general comprehensive understanding of *A. mellife*ra micronutritional needs [34,52]. Indeed, pollen, which is rich in micronutrients [10], appears to be consistently preferred over artificial substitutes [33,82], adding evidence for maintaining diverse floral resources bee nutrition. Nonetheless, supplementation is a needed resource for beekeepers in times of need, and the multivitamin dosages here, although not detrimental [12], do not appear to be the correct ones for caged bees.

A fundamental component in sustaining bees under controlled conditions for biologically relevant data (i.e., translatable data to real-hive scenarios) is a study system designed to offer a proper environment with adequate nutrition while closely replicating natural field conditions (e.g., trophallaxis, cleansing flights, brood and queen pheromones, etc, [15]. Indeed, decades-old efforts have been made to study *A. mellifera* in controlled settings [85,86], and different materials have been used, such as wooden cages wire mesh for ventilation with 50 workers/cage [87], single-use plastic cups (384 cm^3^) with ≈ 80 integrated holes (⌀ 5 mm) for ventilation and 60 workers/cage [88], or smaller polystyrene containers (177 cm^3^) with nylon mesh for ventilation and 15 workers/cage [89]. Recent efforts for allowing cleansing flights in laboratory conditions have also been used [49]. Indeed, standardized practices, as used here, have been established [57], and do offer the advantage of higher replicate numbers, thus providing robust statistical data. However, field colonies would be the best option, but offer severe drawbacks, including (but not limited to) time constraints, stochastic environmental conditions, space, elevated costs, and difficultly of achieving high enough sample numbers for robust conclusions. Therefore, an alternative solution that would allow the controlled conditions of the laboratory while simulating real-hive conditions (i.e., colony pheromones, wax, honey, bee bread, cleansing flights, [15] would be optimal. Here, we propose that Apidea mating hives [90] made of 20 cm thick styrofoam and are compact in size (24 cm x 15 cm x 17 cm), with 5 fames, bottom board, and top-feeder as a viable solution to try. Our in-house data suggests they readily house 100g of bees (≈ 1000 workers), with the straightforward possibility of expanding the brood chamber if desired. Caged-bees do not defecate [91], hindering the clearance of expelling amassed waste in their hindguts, a known trigger of premature death in honey bees [74]. Apidae hives offer entrances that can be opened and closed, allowing easy hive manipulation and opening the possibility of transport to outdoor netted areas where cleansing flights (e.g., waste excretion) on warmer days can take place, or in warm indoor settings [49]. Furthermore, the use of a climate chamber, such as the one used here (Memmert^©^ HPP 750) allows for stable humidity and temperature control, with adjustable settings of 4^o^C - 10^o^C. We argue this would be a compromise for the drawbacks of full-colony experiments, while maintaining controlled laboratory conditions, and lastly, yielding data that would be easier to translate for outdoor use.

The results of this study reinforce the significant role of pollen in *A. mellifera* diets, with clear impacts on their weight and longevity. Despite the lack of observed benefits from the multivitamin supplementation, the findings nonetheless offer a starting point for future range-finding studies aimed at discovering better vitamin dosing. Furthermore, the observations here bolster the need of future research to focus efforts on refining laboratory conditions to better mimic real-hive environments, such as the use of Apidae boxes kept in controlled conditions. Indeed, such data would derive more translatable conclusions, particularly for long-lived “winter” bees, with the ultimate goal of improving apiculture practices and ensuring the long-term viability of managed *A. mellifera* colonies.

## Supporting information

S1 Fig Kaplan-Meier survival curves from adult summer (left) and winter (right) *Apis mellifera* workers from eight treatments: Sucrose, Sucrose + Pollen, Vitamin 1, Vitamin 1 + Pollen, Vitamin 2, Vitamin 2 + Pollen, Vitamin 3, Vitamin 3 + Pollen. (N = 208 workers/treatment group/season, N = 8 replicates/treatment, N = 26 bees/treatment, N = 1664 total workers/season. Each line and color represent a different treatment. Dotted lines indicate groups supplemented ad libitum pollen. Significant differences between survival outcomes of the experimental workers are indicated by letters based on log rank tests and Bonferroni p-adjusted values (Ps < 0.05).
(DOCX)

S1 Table Summary statistics of pollen and sucrose consumption from Apis mellifera adult workers. Sucrose consumption was measured for Sucrose, Sucrose + Pollen, Vitamin 1, Vitamin 1 + Pollen, Vitamin 2, Vitamin 2 + Pollen, Vitamin 3, Vitamin 3 + Pollen (N = 8), and pollen consumption was additionally measured for: Sucrose + Pollen, Vitamin 1 + Pollen, Vitamin 2 + Pollen, and Vitamin 3 + Pollen. Measurements are displayed in milligrams (mg) were taken with both summer and winter workers. Minimum and maximum values, as well as means, std. deviations, std. errors, 95% CI are displayed.
(DOCX)

S2 Table Linear model summary output of summer sucrose consumption (√mg), winter sucrose consumption (√mg), and pollen-to-carbohydrate (P:C) ratio (%) of adult Apis mellifera workers from two different seasons: summer and winter (n = 2). Fixed Factors, model estimates, standard error, t-values, and p-values, season, and units are displayed.
(DOCX)

S3 Table Summary statistics of dry weight from Apis mellifera adult workers subject to one of eight treatments: Sucrose, Sucrose + Pollen, Vitamin 1, Vitamin 1 + Pollen, Vitamin 2, Vitamin 2 + Pollen, Vitamin 3, Vitamin 3 + Pollen (N = 8). Measurements are displayed in milligrams (mg) were taken with both summer and winter workers. Minimum and maximum values, as well as means, 1^st^ and 3^rd^ quartiles, inter-quartile range (IQR), are displayed.
(DOCX)

S4 Table Summary statistics of longevity from Apis mellifera adult workers subject to one of eight treatments: Sucrose, Sucrose + Pollen, Vitamin 1, Vitamin 1 + Pollen, Vitamin 2, Vitamin 2 + Pollen, Vitamin 3, Vitamin 3 + Pollen (N = 8). Measurements are displayed in days and were taken with both summer and winter workers. Minimum and maximum values, as well as medians, 1^st^ and 3^rd^ quartiles, inter-quartile range, are displayed.
(DOCX)
