# Supplementary material for: Towards holistic colony feeding: Effects of vitamin supplementation on summer and winter honey bee workers, Apis mellifera L
Source: PLoS One. 2025 Aug 28;20(8):e0328626. doi: 10.1371/journal.pone.0328626 (PMC12393766; doi:10.1371/journal.pone.0328626)
Supplement: S1 Fig — (N = 208 workers/treatment group/season, N = 8 replicates/treatment, N = 26 bees/treatment, N = 1664 total workers/season. Each line and color represent a different treatment. Dotted lines indicate groups supplemented ad libitum pollen. Significant differences between survival outcomes of the experimental workers are indicated by letters based on log rank tests and Bonferroni p-adjusted values (Ps < 0.05). (DOCX) [file pone.0328626.s001.docx]

**Towards holistic colony feeding: effects of vitamin supplementation on summer and winter honeybee workers, *Apis mellifera***

Andrew F. Brown^1*^, Leah Guillaume-Gentil^1^, Johanna Hehl^1^, Stefan Niederer^1^, Gina Retschnig^1^, Peter Neumann^1^

^1^Institute of Bee Health, Vetsuisse Faculty, University of Bern, Schwarzenburgstrasse 161, 3003 Bern, Switzerland

*Correspondence: [andrew.f.brown@outlook.com](mailto:andrew.f.brown@outlook.com)

**Supplementary Information**

**Figures**

**
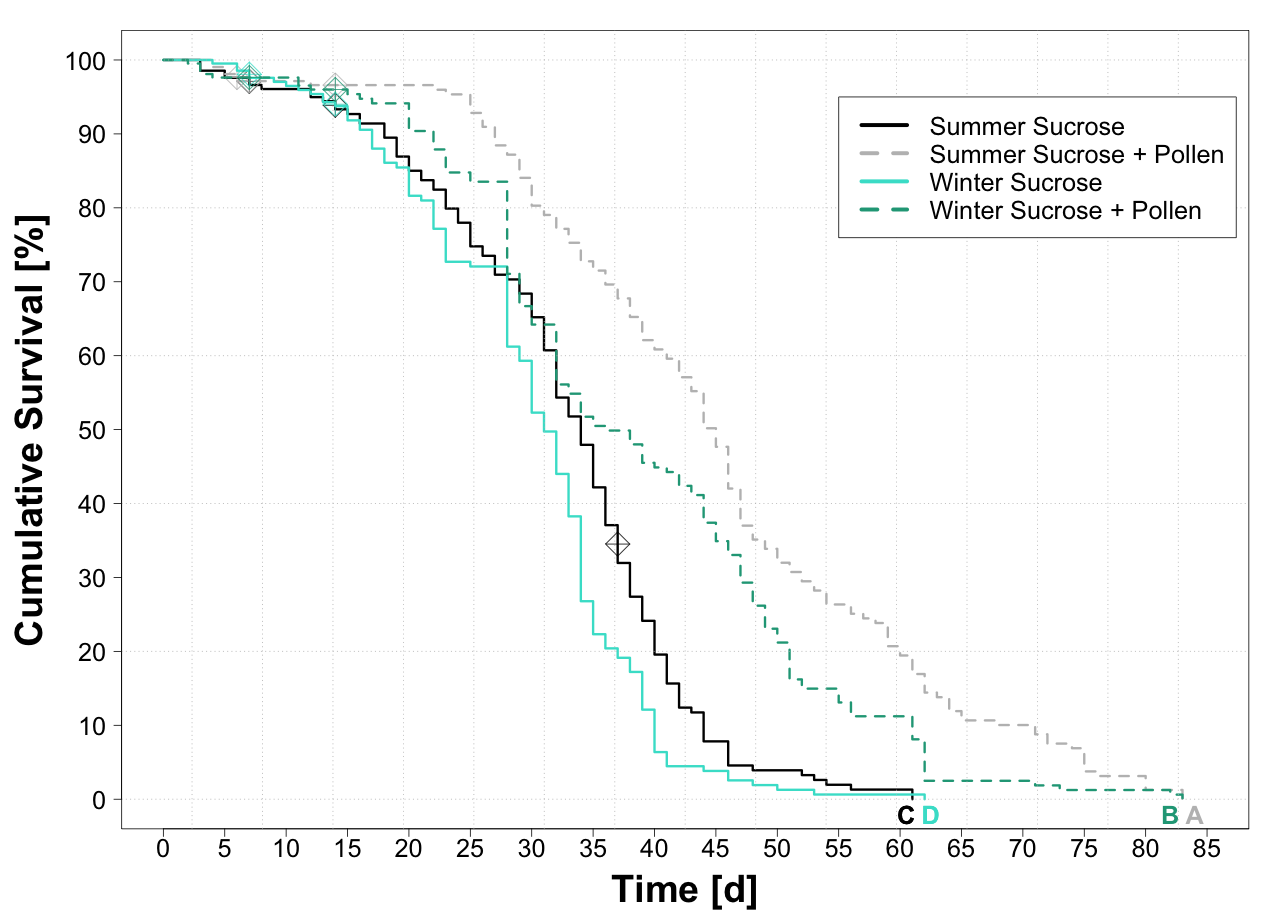
**

**Fig S1**: Kaplan-Meier survival curves from adult summer (left) and winter (right) Apis mellifera workers from eight treatments: Sucrose, Sucrose + Pollen, Vitamin 1, Vitamin 1 + Pollen, Vitamin 2, Vitamin 2 + Pollen, Vitamin 3, Vitamin 3 + Pollen. (N=208 workers/treatment group/season, N=8 replicates/treatment, N=26 bees/treatment, N=1664 total workers/season. Each line and color represent a different treatment. Dotted lines indicate groups supplemented ad libitum pollen. Significant differences between survival outcomes of the experimental workers are indicated by letters based on log rank tests and Bonferroni p-adjusted values (Ps<0.05).
