# Supplementary material for: Towards holistic colony feeding: Effects of vitamin supplementation on summer and winter honey bee workers, Apis mellifera L
Source: PLoS One. 2025 Aug 28;20(8):e0328626. doi: 10.1371/journal.pone.0328626 (PMC12393766; doi:10.1371/journal.pone.0328626)
Supplement: S1 Table — Sucrose consumption was measured for Sucrose, Sucrose + Pollen, Vitamin 1, Vitamin 1 + Pollen, Vitamin 2, Vitamin 2 + Pollen, Vitamin 3, Vitamin 3 + Pollen (N = 8), and pollen consumption was additionally measured for: Sucrose + Pollen, Vitamin 1 + Pollen, Vitamin 2 + Pollen, and Vitamin 3 + Pollen. Measurements are displayed in milligrams (mg) were taken with both summer and winter workers. Minimum and maximum values, as well as means, std. deviations, std. errors, 95% CI are displayed. (DOCX) [file pone.0328626.s002.docx]

**Towards holistic colony feeding: effects of vitamin supplementation on summer and winter honeybee workers, *Apis mellifera***

Andrew F. Brown^1*^, Leah Guillaume-Gentil^1^, Johanna Hehl^1^, Stefan Niederer^1^, Gina Retschnig^1^, Peter Neumann^1^

^1^Institute of Bee Health, Vetsuisse Faculty, University of Bern, Schwarzenburgstrasse 161, 3003 Bern, Switzerland

*Correspondence: [andrew.f.brown@outlook.com](mailto:andrew.f.brown@outlook.com)

**Supplementary Information**

| **Treatment** | **Minimum** | **Maximum** | **Mean** | **Std. Deviation** | **Std. Error** | **95%CI ±** | **Consumption** | **Season** |
| --- | --- | --- | --- | --- | --- | --- | --- | --- |
| Sucrose | 12.7 | 69.1 | 32.6 | 11.7 | 0.902 | 1.8 | Sucrose | Summer |
| Sucrose + Pollen | 8.11 | 59.9 | 29.8 | 10.6 | 0.826 | 1.7 | Sucrose | Summer |
| Vitamin 1 | 13.1 | 64.4 | 32.4 | 10.8 | 0.837 | 1.6 | Sucrose | Summer |
| Vitamin 1 + Pollen | 8.81 | 62.1 | 29 | 10.2 | 0.788 | 1.6 | Sucrose | Summer |
| Vitamin 2 | 7.56 | 76.2 | 31.8 | 11.5 | 0.886 | 1.8 | Sucrose | Summer |
| Vitamin 2 + Pollen | 9.27 | 60.8 | 32.4 | 11.6 | 0.894 | 1.7 | Sucrose | Summer |
| Vitamin 3 | 11.3 | 63.9 | 30.6 | 11.5 | 0.889 | 1.8 | Sucrose | Summer |
| Vitamin 3 + Pollen | 4.05 | 80.6 | 31.7 | 12 | 0.938 | 1.9 | Sucrose | Summer |
|  |  |  |  |  |  |  |  |  |
| Sucrose | 4.21 | 65.7 | 30.8 | 11.8 | 0.860 | 1.7 | Sucrose | Winter |
| Sucrose + Pollen | 7.98 | 85.1 | 34.1 | 13.7 | 0.996 | 2 | Sucrose | Winter |
| Vitamin 1 | 3.6 | 64.8 | 31.9 | 11.5 | 0.828 | 1.6 | Sucrose | Winter |
| Vitamin 1 + Pollen | 7.16 | 93.7 | 34.1 | 14.5 | 1.05 | 2.1 | Sucrose | Winter |
| Vitamin 2 | 7.05 | 55 | 30.2 | 10.8 | 0.78 | 1.6 | Sucrose | Winter |
| Vitamin 2 + Pollen | 8.38 | 80.1 | 34.6 | 14.5 | 1.06 | 2.1 | Sucrose | Winter |
| Vitamin 3 | 8.49 | 57.5 | 29.9 | 10.6 | 0.771 | 1.5 | Sucrose | Winter |
| Vitamin 3 + Pollen | 9.93 | 69.1 | 32.6 | 13.9 | 1.04 | 2 | Sucrose | Winter |
|  |  |  |  |  |  |  |  |  |
| Sucrose + Pollen | 0.348 | 6.98 | 2.49 | 1.8 | 0.137 | 0.27 | Pollen | Summer |
| Vitamin 1 + Pollen | 0.47 | 7.38 | 2.49 | 1.83 | 0.14 | 0.27 | Pollen | Summer |
| Vitamin 2 + Pollen | 0.0548 | 7.81 | 2.51 | 1.86 | 0.142 | 0.28 | Pollen | Summer |
| Vitamin 3 + Pollen | 0.313 | 7.25 | 2.39 | 1.88 | 0.144 | 0.29 | Pollen | Summer |
|  |  |  |  |  |  |  |  |  |
| Sucrose + Pollen | 0.274 | 8.12 | 2.36 | 1.9 | 0.138 | 0.27 | Pollen | Winter |
| Vitamin 1 + Pollen | 0.3 | 9.47 | 2.45 | 1.93 | 0.14 | 0.27 | Pollen | Winter |
| Vitamin 2 + Pollen | 0.11 | 8.56 | 2.59 | 1.96 | 0.143 | 0.29 | Pollen | Winter |
| Vitamin 3 + Pollen | 0.117 | 11.1 | 2.7 | 2.03 | 0.152 | 0.3 | Pollen | Winter |

**S1 Table S1**: Summary statistics of pollen and sucrose consumption from Apis mellifera adult workers. Sucrose consumption was measured for Sucrose, Sucrose + Pollen, Vitamin 1, Vitamin 1 + Pollen, Vitamin 2, Vitamin 2 + Pollen, Vitamin 3, Vitamin 3 + Pollen (N=8), and pollen consumption was additionally measured for: Sucrose + Pollen, Vitamin 1 + Pollen, Vitamin 2 + Pollen, and Vitamin 3 + Pollen. Measurements are displayed in milligrams (mg) were taken with both summer and winter workers. Minimum and maximum values, as well as means, std. deviations, std. errors, 95% CI are displayed.
