# Supplementary material for: Towards holistic colony feeding: Effects of vitamin supplementation on summer and winter honey bee workers, Apis mellifera L
Source: PLoS One. 2025 Aug 28;20(8):e0328626. doi: 10.1371/journal.pone.0328626 (PMC12393766; doi:10.1371/journal.pone.0328626)
Supplement: S2 Table — Fixed Factors, model estimates, standard error, t-values, and p-values, season, and units are displayed. (DOCX) [file pone.0328626.s003.docx]

**Towards holistic colony feeding: effects of vitamin supplementation on summer and winter honeybee workers, *Apis mellifera***

Andrew F. Brown^1*^, Leah Guillaume-Gentil^1^, Johanna Hehl^1^, Stefan Niederer^1^, Gina Retschnig^1^, Peter Neumann^1^

^1^Institute of Bee Health, Vetsuisse Faculty, University of Bern, Schwarzenburgstrasse 161, 3003 Bern, Switzerland

*Correspondence: [andrew.f.brown@outlook.com](mailto:andrew.f.brown@outlook.com)

**Supplementary Information**

**SI Table S2:** Linear model summary output of summer sucrose consumption ($\surd mg$), winter sucrose consumption ($\surd mg$), and pollen-to-carbohydrate (P:C) ratio (%) of adult Apis mellifera workers from two different seasons: summer and winter (n=2). Fixed Factors, model estimates, standard error, t-values, and p-values, season, and units are displayed.

| **Fixed Factors** | **Estimates** | **Std. Error** | **T-Value** | **P-Value** | **Season** | **units** |
| --- | --- | --- | --- | --- | --- | --- |
| (Intercept: Sucrose) | 4.778 | 0.110 | 43.569 | **<0.001** | summer | milligrams |
| Sucrose + Pollen | -0.264 | 0.106 | -2.492 | **0.013** | summer | milligrams |
| Vitamin 1 | -0.006 | 0.106 | -0.059 | 0.953 | summer | milligrams |
| Vitamin 1 + Pollen | -0.327 | 0.106 | -3.095 | **0.002** | summer | milligrams |
| Vitamin 2 | -0.072 | 0.106 | -0.682 | 0.495 | summer | milligrams |
| Vitamin 2 + Pollen | -0.025 | 0.106 | -0.241 | 0.810 | summer | milligrams |
| Vitamin 3 | -0.189 | 0.106 | -1.788 | 0.074 | summer | milligrams |
| Vitamin 3 + Pollen | -0.099 | 0.106 | -0.935 | 0.350 | summer | milligrams |
| Day^1^ | 0.187 | 0.017 | 11.250 | **<0.001** | summer | milligrams |
| Day^2^ | -0.007 | 0.001 | -10.949 | **<0.001** | summer | milligrams |
|  |  |  |  |  |  |  |
| (Intercept: Sucrose) | 4.037 | 0.111 | 36.355 | **<0.001** | winter | milligrams |
| Sucrose + Pollen | 0.273 | 0.106 | 2.577 | **0.010** | winter | milligrams |
| Vitamin 1 | 0.104 | 0.105 | 0.984 | 0.325 | winter | milligrams |
| Vitamin 1 + Pollen | 0.259 | 0.106 | 2.456 | **0.014** | winter | milligrams |
| Vitamin 2 | -0.050 | 0.105 | -0.471 | 0.638 | winter | milligrams |
| Vitamin 2 + Pollen | 0.305 | 0.106 | 2.877 | **0.004** | winter | milligrams |
| Vitamin 3 | -0.061 | 0.106 | -0.575 | 0.566 | winter | milligrams |
| Vitamin 3 + Pollen | 0.139 | 0.107 | 1.293 | 0.196 | winter | milligrams |
| Day^1^ | 0.305 | 0.016 | 19.241 | **<0.001** | winter | milligrams |
| Day^2^ | -0.012 | 0.001 | -19.129 | **<0.001** | winter | milligrams |
|  |  |  |  |  |  |  |
| (Intercept: Summer ) | 38.227 | 0.784 | 48.762 | **<0.001** | summer:winter | P:C ratio |
| Winter | 8.745 | 1.097 | 7.969 | **<0.001** | summer:winter | P:C ratio |
| Day^1^ | -4.650 | 0.148 | -31.347 | **<0.001** | summer:winter | P:C ratio |
| Day^2^ | 0.144 | 0.006 | 25.108 | **<0.001** | summer:winter | P:C ratio |
| Winter * Day^1^ | -1.263 | 0.206 | -6.134 | **<0.001** | summer:winter | P:C ratio |
| Winter * Day^2^ | 0.042 | 0.008 | 5.262 | **<0.001** | summer:winter | P:C ratio |
