# Supplementary material for: Towards holistic colony feeding: Effects of vitamin supplementation on summer and winter honey bee workers, Apis mellifera L
Source: PLoS One. 2025 Aug 28;20(8):e0328626. doi: 10.1371/journal.pone.0328626 (PMC12393766; doi:10.1371/journal.pone.0328626)
Supplement: S3 Table — Measurements are displayed in milligrams (mg) were taken with both summer and winter workers. Minimum and maximum values, as well as means, 1st and 3rd quartiles, inter-quartile range (IQR), are displayed. (DOCX) [file pone.0328626.s004.docx]

**Towards holistic colony feeding: effects of vitamin supplementation on summer and winter honeybee workers, *Apis mellifera***

Andrew F. Brown^1*^, Leah Guillaume-Gentil^1^, Johanna Hehl^1^, Stefan Niederer^1^, Gina Retschnig^1^, Peter Neumann^1^

^1^Institute of Bee Health, Vetsuisse Faculty, University of Bern, Schwarzenburgstrasse 161, 3003 Bern, Switzerland

*Correspondence: [andrew.f.brown@outlook.com](mailto:andrew.f.brown@outlook.com)

**Supplementary Information**

**SI Table S3**: Summary statistics of dry weight from Apis mellifera adult workers subject to one of eight treatments: Sucrose, Sucrose + Pollen, Vitamin 1, Vitamin 1 + Pollen, Vitamin 2, Vitamin 2 + Pollen, Vitamin 3, Vitamin 3 + Pollen (N=8). Measurements are displayed in milligrams (mg) were taken with both summer and winter workers. Minimum and maximum values, as well as means, 1^st^ and 3^rd^ quartiles, inter-quartile range (IQR), are displayed.

| **Treatment** | **Minimum** | **Maximum** | **1st Quartile** | **Median** | **3rd Quartile** | **IQR** | **Season** |
| --- | --- | --- | --- | --- | --- | --- | --- |
| Sucrose | 17.7 | 58.6 | 22.3 | 25.6 | 28.8 | 6.57 | Summer |
| Sucrose + Pollen | 20 | 60.4 | 34 | 38.6 | 41.8 | 7.75 | Summer |
| Vitamin 1 | 18.3 | 44.2 | 20.7 | 23.8 | 25.6 | 4.95 | Summer |
| Vitamin 1 + Pollen | 25.9 | 50.6 | 36.6 | 40.4 | 43.3 | 6.65 | Summer |
| Vitamin 2 | 18.2 | 46.8 | 21.9 | 24.6 | 28.5 | 6.6 | Summer |
| Vitamin 2 + Pollen | 31 | 54.7 | 37.2 | 39.8 | 41.9 | 4.7 | Summer |
| Vitamin 3 | 17.3 | 54.4 | 21.9 | 24.2 | 27.5 | 5.57 | Summer |
| Vitamin 3 + Pollen | 26.3 | 48.7 | 37.1 | 39.2 | 43 | 5.85 | Summer |
|  |  |  |  |  |  |  |  |
| Sucrose | 19.8 | 51.4 | 22.4 | 24.6 | 26.8 | 4.4 | Winter |
| Sucrose + Pollen | 22.7 | 53.1 | 33.5 | 37.2 | 39.8 | 6.25 | Winter |
| Vitamin 1 | 19.7 | 45.2 | 21.8 | 23.8 | 27 | 5.18 | Winter |
| Vitamin 1 + Pollen | 23.6 | 64.2 | 35.9 | 39.3 | 42.3 | 6.4 | Winter |
| Vitamin 2 | 18.3 | 43 | 21.9 | 23 | 26.8 | 4.93 | Winter |
| Vitamin 2 + Pollen | 22.7 | 53.5 | 31.5 | 38.2 | 40.6 | 9.15 | Winter |
| Vitamin 3 | 18.7 | 55.7 | 21.3 | 24.4 | 27.7 | 6.38 | Winter |
| Vitamin 3 + Pollen | 21.4 | 51.1 | 32 | 38.6 | 42 | 10 | Winter |
