# Supplementary material for: Towards holistic colony feeding: Effects of vitamin supplementation on summer and winter honey bee workers, Apis mellifera L
Source: PLoS One. 2025 Aug 28;20(8):e0328626. doi: 10.1371/journal.pone.0328626 (PMC12393766; doi:10.1371/journal.pone.0328626)
Supplement: S4 Table — Measurements are displayed in days and were taken with both summer and winter workers. Minimum and maximum values, as well as medians, 1st and 3rd quartiles, inter-quartile range, are displayed. (DOCX) [file pone.0328626.s005.docx]

**Towards holistic colony feeding: effects of vitamin supplementation on summer and winter honeybee workers, *Apis mellifera***

Andrew F. Brown^1*^, Leah Guillaume-Gentil^1^, Johanna Hehl^1^, Stefan Niederer^1^, Gina Retschnig^1^, Peter Neumann^1^

^1^Institute of Bee Health, Vetsuisse Faculty, University of Bern, Schwarzenburgstrasse 161, 3003 Bern, Switzerland

*Correspondence: [andrew.f.brown@outlook.com](mailto:andrew.f.brown@outlook.com)

**Supplementary Information**

| **Treatment** | **Minimum** | **Maximum** | **1^st^ quartile** | **Median** | **3^rd^ quartile** | **IQR** | **Season** |
| --- | --- | --- | --- | --- | --- | --- | --- |
| Sucrose | 3 | 61 | 14 | 30 | 37 | 23 | Summer |
| Sucrose + Pollen | 4 | 83 | 14 | 38 | 50 | 36 | Summer |
| Vitamin 1 | 1 | 54 | 20 | 29 | 35 | 15 | Summer |
| Vitamin 1 + Pollen | 2 | 119 | 14 | 35 | 55 | 41 | Summer |
| Vitamin 2 | 2 | 54 | 14 | 31 | 38 | 24 | Summer |
| Vitamin 2 + Pollen | 1 | 87 | 14 | 32 | 42 | 28 | Summer |
| Vitamin 3 | 2 | 57 | 15 | 30 | 36 | 21 | Summer |
| Vitamin 3 + Pollen | 1 | 73 | 14 | 32 | 44 | 30 | Summer |
|  |  |  |  |  |  |  |  |
| Sucrose | 4 | 62 | 14 | 28 | 34 | 20 | Winter |
| Sucrose + Pollen | 2 | 83 | 14 | 30 | 47 | 33 | Winter |
| Vitamin 1 | 1 | 55 | 14 | 28 | 35 | 21 | Winter |
| Vitamin 1 + Pollen | 1 | 90 | 14 | 32 | 45 | 31 | Winter |
| Vitamin 2 | 2 | 56 | 14 | 28 | 34 | 20 | Winter |
| Vitamin 2 + Pollen | 3 | 82 | 14 | 32 | 46 | 32 | Winter |
| Vitamin 3 | 2 | 51 | 14 | 28 | 34 | 20 | Winter |
| Vitamin 3 + Pollen | 1 | 90 | 14 | 28 | 41 | 27 | Winter |

**SI Table S4**: Summary statistics of longevity from Apis mellifera adult workers subject to one of eight treatments: Sucrose, Sucrose + Pollen, Vitamin 1, Vitamin 1 + Pollen, Vitamin 2, Vitamin 2 + Pollen, Vitamin 3, Vitamin 3 + Pollen (N=8). Measurements are displayed in days and were taken with both summer and winter workers. Minimum and maximum values, as well as medians, 1^st^ and 3^rd^ quartiles, inter-quartile range, are displayed.
